# Vaccination

**Published:** 1882-03

**Authors:** 


### VACCINATION.

Prejudice, which sees what it pleases,
Cannot see what is plain.—*Aubrey de Vere.*

To most persons the question of vaccination doubtless appears to be a settled one. Yet it is not. For while grand results are claimed for the practice, and these supported by unlimited statistical evidence and personal authority, there is a very respectable minority, who condemn the practice as wholly pernicious and thorougly useless. Therefore, a brief *resumé* of the main points advanced in the discussion of vaccination will not be inappropriate at this time. Very briefly put, the chief results alleged to be obtained, are:

1. Vaccination, *well performed*, protects the subject from variolous infection. (Great stress is laid on its being thoroughly well performed with the Jennerian vaccine lymph).

2. Should variola, be taken, vaccination renders its course less virulent, and almost precludes a fatal issue.

3. Vaccination has materially diminished the frequency of epidemics of variola.*

4. Vaccination has raised the standard of health, i. e., lowered the general rate of mortality. This last plea rose from the idea that small-pox so lowered the vitality of the patient that he was apt after suffering from it to go into "decline," or fall an easy prey to other diseases. (The death rate in England before the discovery of vaccination was 21.80 per 1000; after vaccination came into vogue it was 22!) All the sealleged advantages depend on the question does vaccination protect from variola? If this be decided in the affirmative then these results must be acknowledged.

We have no desire to argue this question; much has been said to prove it; statistics innumerable have been compiled to show it. These arguments and statistics are so familiar to all that we shall pass them over, quoting none. We merely say that if figures ever proved any fact, they prove the unquestionable value of vaccination.

---

* "Small-pox attained its maximum mortality after inoculation was introduced. The annual deaths from small-pox were 2,323, 1760-79; in the next twenty years they declined to 1,740; this disease therefore began to grow less fatal before vaccination was discovered; indicating together with the diminution of fever, the general improvement in health then taking place."—Dr. Farr on Epidemics in M'Cullock's *Descriptive and Statistical Account* of the British Empire, 4th ed. Vol. II.

If personal authority, long lists of brilliant names, unanimously advocating a measure, prove anything, then the great value of vaccination is shown incontestably. But these are not infallible proofs, for those same figures, those same illustrious men, once proved as positively and swore as sincerely in favor of *inoculation*, as they now do in favor of vaccination! Yet, at a later period the British Parliament was persuaded to interdict inoculation as an injurious practice.

Again, these same illustrious men—such as Henry Lee, Acton, and Langston Parker, renowned syphilographers, united with professional vaccinators, of England, such as Marston, Leese, and Tomkins—men who vaccinated hundreds of thousands—these all declared that no syphilis or other dangerous disorder had ever been inoculated through vaccination. Yet, in spite of this unanimous declaration against syphilization, etc., we find "arm to arm" vaccination almost abandoned in favor of vaccination from animal lymph.

And the cases related by Mr. Jonathan Hutchinson compelled Sir Thomas Watson to say (in *Nineteenth Century*, June, 1878), "It is certain that one objection, really formidable, does exist: that the operation (arm to arm vaccination) may impart a hateful and destructive disease (syphilis)." Thus we see the authority of these figures and great men has been impeached and overthrown on two vital questions: (1) inoculation, and (2) syphilization from vaccination. To how much credit then are they entitled when they defend and urge vaccination? Again, we as homœopathists know how these same facts and figures err when they attempt to prove the falsity and weakness of our practice.

We do not decry vaccination from any antipathy to it, but simply desire to point out some of its weak points, and to note some of its positively injurious features. We do this because we feel convinced that the vaccinating lancet is carelessly and dangerously used. We would therefore call attention to some of the injuries it has been charged with. These apply chiefly to the use of human virus in what are known as "arm to arm" vaccinations.

1. It is alleged that vaccination has produced new diseases, such as mental and nervous diseases, erysipelas, etc., and that glandular and cutaneous diseases have been invaccinated.

2. That vaccination has led to an increase in mortality from other diseases, such as fevers and scrofulous diseases.

3. It is denied that vaccination protects one from variola. It is also denied that variola is as infectious as commonly believed; *per contra*, it is affirmed that fresh air, cleanliness, etc., are its best preventives. In this connection, we cannot refrain from quoting the following catechism from a Dr. Wilkinson:

Q. When whooping cough is not rife, to what is it due?
A. Nature.
Q. When scarlatina is not rife, to what is it due?
A. Nature.
Q. When cholera is not rife, to what is it due?
A. Nature.
Q. When variola is not rife, to what is it due?
A. Vaccination.
Q. When other diseases in course of time become mild or die out, to what is it due?
A. Nature.
Q. When variola becomes mild or dies out, to what is it due?
A. Vaccination.

As to hygiene and small-pox, we quote the words of Florence Nightingale: "I have seen with my eyes, and smelt with my nose, small-pox growing up in first specimens; either in close rooms or in over-crowded wards, where it could not, by any possibility, have been caught, but must have begun."

Having very briefly pointed out the alleged virtues and defects of vaccination, let us close with a few words as to how and when this operation should be performed, that those who perform it may do so intelligently. For this purpose, we quote from Dr. Seaton's *Handbook of Vaccination.* Two varieties of vaccine lymph are used, the animal and the human. Animal lymph comes from natural or artificial cow-pox. Cows and horses are liable to a contagious disease, known in the one as cow-pox and in the other as horse-pox.* This disease is now seldom noticed; Jenner and others thought cow-pox was only produced by inoculation from a horse suffering with the "Grease," but this has since been proved erroneous. The animal lymph now used is from the cow-pox produced by artificial inoculation. This may be done:

* Sheep have a disease called sheep-pox; it is more virulent in its course, and its vesicles are flat and not umbilicated as the vesicles of cow-pox. Inoculation prevents this sheep-pox and lessens its severity.

1. By lymph taken from cows suffering from the natural or inoculated cow-pox.
2. By the lymph from the horse-pox—*equination*.*
3. By lymph, derived originally from a cow or horse, but passed through the human system by vaccination—*retro-vaccination*.
4. By the matter of human variola—*variolation*.

The first method is chiefly used; that is, the heifers are inoculated with the lymph of cow-pox and the lymph, taken from vesicles so produced, is used for vaccinating subjects. The human lymph has been to a great degree discarded, for fear of inoculation of syphilis, scrofula, etc. The liability to this danger was at first denied, but is now very generally admitted by the profession and greatly feared by the laity. It is said that cow-lymph does not "take" so certainly as does the human-lymph.

So much for the lymph to be used. As to time and manner of its use, Dr. Seaton says: In vaccinating special attention must be given (*a*) to the state of the person to be vaccinated, (*b*) to the proper selection of the lymph to be used, and (*c*) to the proper and thorough insertion of the lymph.

The person to be vaccinated should be in good health, free from acute or chronic disease, as it may interfere with or aggravate the operation. Healthy children may be operated on when four or six weeks old. If variola be very prevalent in a neighborhood, or in the house, then it is advisable to vaccinate immediately, regardless of present state of health. The lymph used should be taken about the eighth day, *before the appearance of the "areola,"* from thoroughly characteristic vesicles. If it be human lymph, it should be taken from persons known to be *healthy*. Children are best; those "of dark complexion, not too florid, with thick, smooth, clear skin, are the best lymph producers." If animal, the healthiest are of course selected. As to the insertion of the lymph, two or three punctures may be made, or two or three small abraded surfaces made, and the lymph well rubbed in; little blood should be drawn, only a mere reddening of the skin is needed. These spots should be an half inch or so apart, so that each puncture forms a separate sore, and

---

* Concerning this Jenner said: "Although the absorption of matter form sores on the heels of horses, secures, or nearly secures, the system from variolous infection, yet it is possible that this cannot be entirely relied upon, until a disease has been generated by morbid matter from the horse or the nipple of the cow, and passed through that medium to the human subject."

leaves a *distinct*, well-developed mark. Some advise that two or three punctures be made, some on one arm and some on the other. If the dried lymph be used, it is softened by moistening it with a little *cold* water. After the operation, the arm should be protected from rubbing, etc., lest undue inflammation be started. The sore should be examined on the eighth day to see that a perfectly characteristic vesicle has been produced. At this date the vesicle should appear round, plump and decidedly pearl-colored, distended with clear lymph; the elevation of its margin and the depression of its centre are very marked. A ring of inflammation, termed areola, begins to form about the base of this vesicle, at this date, or sometimes a few hours earlier. The vesicle and the areola together continue to spread for the next two (9th and 10th) days. The areola is circular and may reach a diameter of two inches. With this we may have swelling of arm, of glands, fever, restlessness and diarrhœa. After the tenth day the areola begins to fade, the vesicle to dry, and by the sixteenth day a hard brown scab is formed, which soon falls off and leaves the characteristic mark.

We have endeavored very briefly to point out the danger of vaccination in order to show that it is by no means a simple, harmless operation. Physicians should perform it with a care and watchfulness, which we fear they do not always render.

